# Video-based training of situation awareness enhances minimally invasive surgical performance: a randomized controlled trial

**DOI:** 10.1007/s00464-023-10006-z

**Published:** 2023-04-14

**Authors:** Karl-Friedrich Kowalewski, Laura Seifert, Laura Kohlhas, Mona Wanda Schmidt, Seher Ali, Carolyn Fan, Karl Felix Köppinger, Beat Peter Müller-Stich, Felix Nickel

**Affiliations:** 1 Department of General, Visceral, and Transplantation Surgery, University of Heidelberg, Im Neuenheimer Feld 420, 69120 Heidelberg, Germany; 2 Department of Urology, University Medical Center Mannheim, Heidelberg University, Mannheim, Germany; 3 Department of Medical Biometry, University of Heidelberg, Im Neuenheimer Feld 130.3, 69120 Heidelberg, Germany; 4 Department of Gynecology and Obstetrics, University Medical Center of Johannes Gutenberg University, Mainz, Germany; 5 Department of Surgery, Clarunis University Center for Gastrointestinal and Liver Disease, University Hospital and St. Clara Hospital Basel, Basel, Switzerland; 6 Department of General, Visceral, and Thoracic Surgery, University Medical Center Hamburg-Eppendorf, Martinistraße 52, 20246 Hamburg, Germany

**Keywords:** Situation(al) awareness, Minimally invasive surgery, Laparoscopic training, Surgical education, Cholecystectomy

## Abstract

**Background:**

Many training curricula were introduced to deal with the challenges that minimally invasive surgery (MIS) presents to the surgeon. Situational awareness (SA) is the ability to process information effectively. It depends on general cognitive abilities and can be divided into three steps: perceiving cues, linking cues to knowledge and understanding their relevance, and predicting possible outcomes. Good SA is crucial to predict and avoid complications and respond efficiently. This study aimed to introduce the concept of SA into laparoscopic training.

**Methods:**

This is a prospective, randomized, controlled study conducted at the MIS Training Center of Heidelberg University Hospital. Video sessions showing the steps of the laparoscopic cholecystectomy (LC) were used for cognitive training. The intervention group trained SA with interposed questions inserted into the video clips. The identical video clips, without questions, were presented to the control group. Performance was assessed with validated scores such as the Objective Structured Assessment of Technical Skills (OSATS) during LC.

**Results:**

72 participants were enrolled of which 61 were included in the statistical analysis. The SA-group performed LC significantly better (OSATS-Score SA: 67.0 ± 11.5 versus control: 59.1 ± 14.0, *p* value = 0.034) and with less errors (error score SA: 3.5 ± 1.9 versus control: 4.7 ± 2.0, *p* value = 0.027). No difference in the time taken to complete the procedure was found. The benefit assessment analysis showed no difference between the groups in terms of perceived learning effect, concentration, or expediency. However, most of the control group indicated retrospectively that they believed they would have benefitted from the intervention.

**Conclusion:**

This study suggests that video-based SA training for laparoscopic novices has a positive impact on performance and error rate. SA training should thus be included as one aspect besides simulation and real cases in a multimodal curriculum to improve the efficiency of laparoscopic surgical skills training.

**Supplementary Information:**

The online version contains supplementary material available at 10.1007/s00464-023-10006-z.

Minimally invasive surgery (MIS) has replaced open procedures as the gold standard in many areas of surgery. This includes simple procedures, such as laparoscopic appendectomy or cholecystectomy [1], but also more complex procedures, like extensive oncological or bariatric surgery. Benefits of MIS include less trauma and postoperative pain, a shorter hospitalization and convalescence time, as well as better cosmetic results [2–4]. These benefits contrast a limited field of vision and mostly two-dimensional imaging, resulting in limited depth perception. Other complicating factors include handling of the laparoscopic instruments due to reduced haptics and limited degrees of freedom [5, 6]. These aspects reciprocate a high demand for psychomotor skills, leading to a prolonged learning curve in MIS [7]. Hands-on experience is essential for surgical education and opportunities can be scarce [8].

The paradigm shift in training methodology toward a competency-based approach is a consequence of increased demands for patient safety. Errors can typically occur more often during surgical training, especially during complex procedures [9]. These errors, along with the prolonged operating time during the learning process, are decisive arguments that learning surgical techniques on patients is outdated. Therefore, several training approaches have been developed to implement MIS training outside the OR [10–14], and structured multi-modality training is beneficial for laparoscopic novices [15].

Currently, resident surgeons usually spend a considerable amount of time assisting and watching experienced surgeons during residency. However, the assisting residents react to the surgeon’s commands and do not necessarily make individual decisions or get involved in planning the next steps, despite being active members of the operating team. Nonetheless, these hours of assisting are of utmost importance. Actively paying attention is crucial to maximizing training effects. This skill of being aware of the current surgical step and anatomical structures at risk will be referred to as “situational awareness” (SA) in this manuscript. In its original definition, SA describes the ability to process information effectively. It depends on general cognitive skills, such as attention, (working) memory and multitasking. SA can be divided into three levels. (1) Perception of key elements in the environment. (2) Recognition of the importance of the key elements and involvement in the current situation. (3) A look into the future and the prediction of a possible outcome for the current situation. Transferred to the OR for a basic procedure, such as laparoscopic cholecystectomy (LC), those three steps could be (1) perception of the preparation status of the cystic duct, (2) recognition that the visualization of the Calot triangle is crucial before clipping and cutting and being aware that anatomical variations of vessels can be present, and (3) if the criteria of the critical view of safety are not met, severe damage of the biliary tree and hepatic blood vessels is at risk. Just as with other mental skills, SA warrants dedicated training to be effective. In addition, it has been shown that SA training for surgeons can reduce errors [16] and could lead to improved performance for residents performing surgery [17]. Therefore, the present study aimed to develop and evaluate a SA training curriculum and to demonstrate that SA may contribute to better surgical performance.

## Materials and methods

### Setting and participants

This study was conducted as a voluntary elective course for medical students in their clinical (3rd to 6th) years at the Medical Faculty of the University of Heidelberg, Germany. The course took place at the MIS Training Center of the Department of General, Visceral and Transplant Surgery. Only laparoscopy-naïve students were included. Students with more than 2 h of prior laparoscopic training were excluded. All participants were informed about the type, extent, and purpose of the study, as well as the possibility to withdraw their consent at any time without disadvantages. SA training was implemented via video clips of recorded LC. Active participation, a fundamental element of SA training, was achieved with interceptive questions (see below). These questions demanded from the participants to distinguish different tissues in the current situation and predict possible outcomes of actions. This type of video-based SA training with interceptive questions was developed following the SA training already described in the literature [18, 19]. To control the impact of the video material itself, the control group viewed the identical video clips without interceptive questions, thus without an active part. Ethical approval was obtained from the Ethics Committee of the Medical Faculty at Heidelberg University (Code S-*436*/2018).

### Study design

This was a prospective, single-center, two-arm, blinded, parallel-group randomized trial. Randomization was performed by a computer-based program and kept safe in sealed opaque and numbered envelopes. There was structured training for the tutors rating the same video clips to ensure inter-rater quality. In order to avoid bias through the tutors by heterogeneity, only two tutors conducted the majority of the trial.

### SA training

**Fig. 1:**
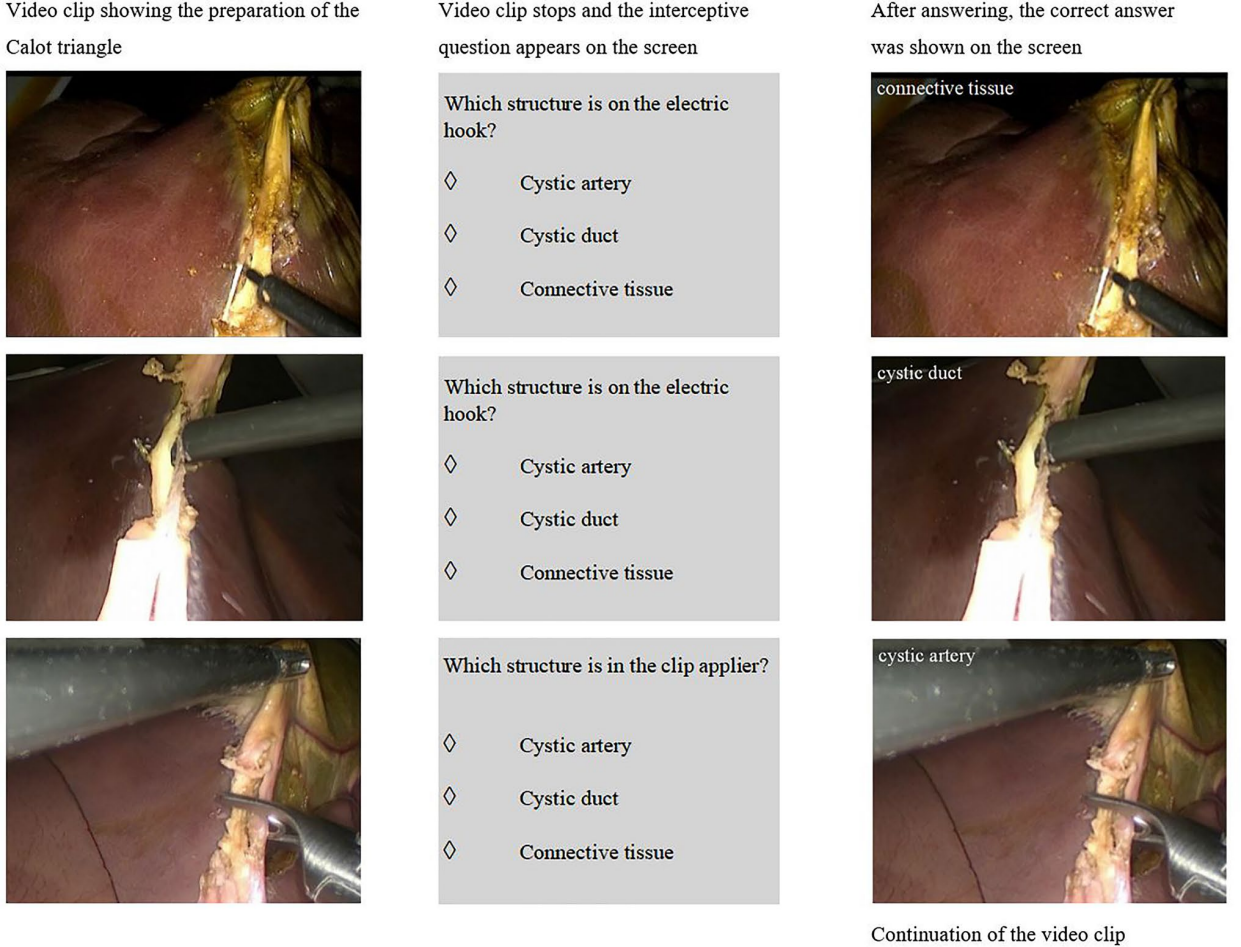
Extract of the intervention training for situational awareness during LC

The intention of the intervention was to train SA. According to Endsley [20], active participation is essential in SA training, which was achieved via the integration of interposed questions into the video clips. Therefore, the video was stopped without warning and a question specific to the current situation appeared (see Fig. 1 and supplementary videos). This method has been described in aviation [21] and in the medical field of anesthesiology [19]. The cognitive training of the control group included video-based training with the identical video clips presented to the intervention group, but without interceptive questions. For the creation of the self-edited video clips, footage from previous studies at the training center was utilized.

The SA training within the curriculum comprised three parts of video sessions. Each video session consisted of two parts:

1. Distinction between the cystic artery, bile duct, and connective tissue during dissection of the Calot triangle.
2. Perception of whether the gallbladder will perforate during its removal from the liver bed.

### Baseline

Prior to training, all participants completed a questionnaire about relevant previous experiences. Questions involved prior experience in the OR, especially in general laparoscopic procedures and open cholecystectomy along with personal characteristics that may be associated with higher performance in (laparoscopic) surgery. Enquired characteristics comprised sports, playing a musical instrument, and playing video games, as done previously[22]. Attention was measured with validated psychological tests (Trail Marking Test (TMT), Frankfurt Attention Inventory 2 (*Frankfurter Aufmerksamkeits-inventar-2: FAIR2*), and Number link test (*Zahlen-Verbindungs-test: ZVT*). Moreover, self-assessment of attention and ability to learn from videos on a scale from 1 to 100 was recorded. In addition, all participants received basic training on a box trainer and the “LAP mentor™.” The baseline of laparoscopic performance was measured with the Heidelberg VR Score [23].

### Pre-test

**Fig. 2:**
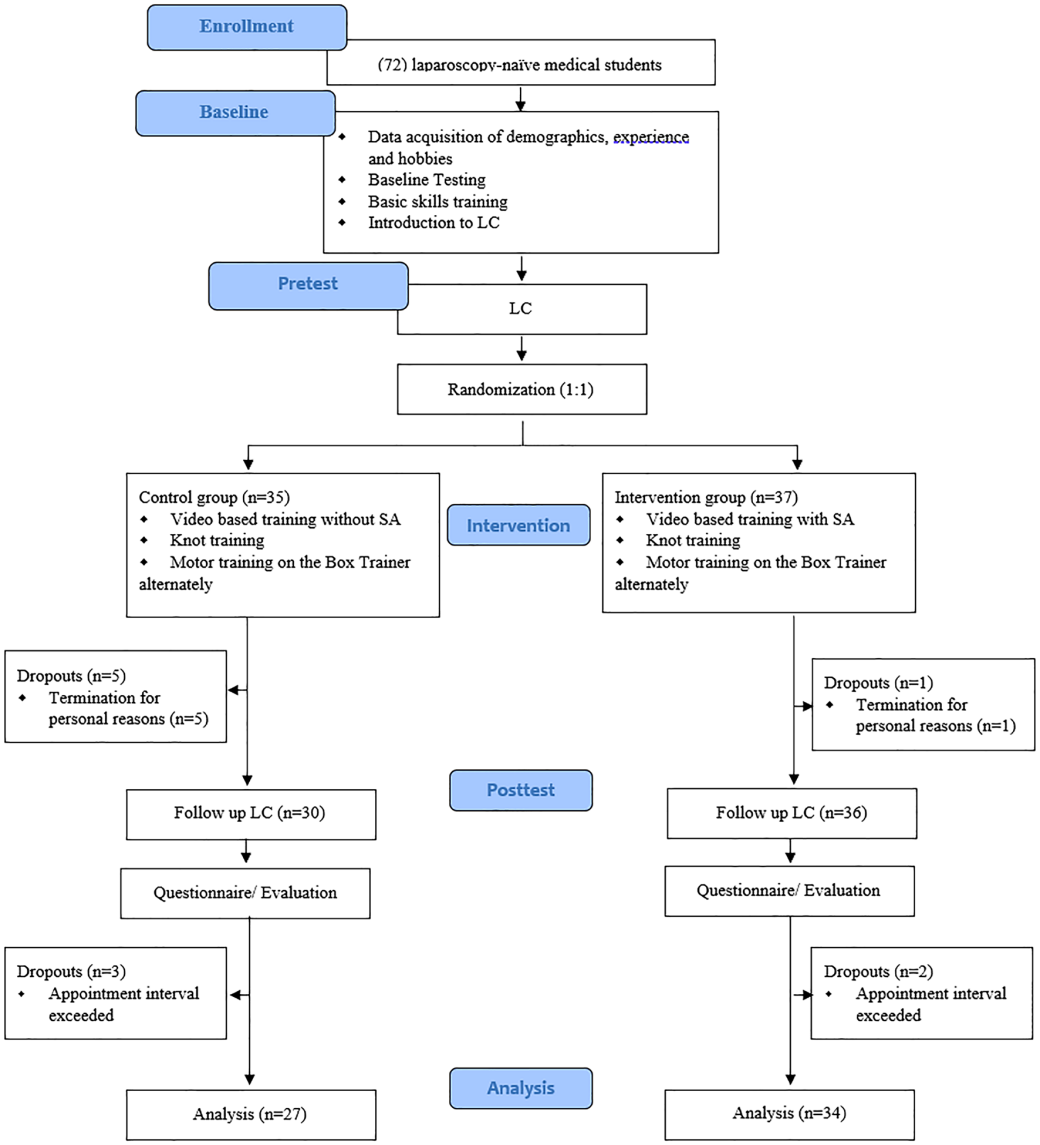
CONSORT Flow diagram of the study design

After an introduction to LC via touch surgery, a validated serious game [24] as well as video- and virtual reality (VR)-based learning, the participants performed their pre-test LC on a porcine cadaver liver model in a box trainer as described earlier [22]. A previous study has shown that it is beneficial to combine serious gaming and VR training to learn the cognitive aspects of LC [24]. The participants were supervised 1:1 but no instructions were given during the procedure. The LC ended either with the completion or after a maximum allowed time of 90 min. Afterward, the participants received feedback. Performance was measured with the Global Operative Assessment of Laparoscopic Skills (GOALS) and the Objective Structured Assessment of Technical Skills (OSATS) as well as an error score. A modified version of the specific technical skill (STS) of the OSATS-Score was used [25]. The difficulty of the procedure was quantified with a visual analog scale (VAS) [26], which included various parameters of the liver and gallbladder, such as the tenderness of the connective tissue, which either facilitated or complicated the dissection of the gallbladder. Next, participants were randomized to receive either SA training or standard video training (see flow chart—Fig. 2).

### Post-test

The post-test was performed under the same conditions as the pre-test. At the end of the training, all participants completed another questionnaire evaluating the training, focusing on cognitive training.

### Study hypothesis

The difference in the OSATS score was defined as the primary outcome.

- H_0_: The OSATS score of the SA-group is equal to or lower than the OSATS score of the control group.
- H_1_: The OSATS score of the SA-group is higher than the OSATS score of the control group.

### Sample size calculation

The sample size determination was calculated with a t test, assumptions from experiences of previous studies and the results of the pilot study. The assumptions made were a significance level of 5%, a standard deviation of 13 and a group difference of 10 points on the OSATS score. Accounting for a drop-out rate of 25%, a sample size of 68 was calculated. Analysis was planned to achieve a power of 80%.

### Statistical analysis

Statistical analysis was performed using R [27]. A *p* value ≤ 0.05 was considered statistically significant.

### Primary outcome

Confirmative testing was performed looking for superiority in the OSATS score at the post-test.

As predefined in the protocol, a stepwise linear regression was used to test the hypothesis. Possible influencing variables were selected beforehand. These were ranked by relevance and significance and gradually removed during model establishment. This was carried out using stepwise regression, identifying the smallest statistically significant model.

### Secondary outcomes

Exploratory analysis was performed by looking for group differences in the post-test of the GOALS Score, the error score, and the total procedure time in minutes. These analyses were performed using the unpaired t tests with equal variances. The individual sub-items of the error score were analyzed with Chi-Square test. Since the study was conducted as a clinical elective course, it was evaluated in detail at the end. General factors, such as supervision, and the different training mediums were evaluated. Furthermore, the cognitive training was evaluated on the perceived expedience of cognitive training, the extent of subjective learning effect, and the level of concentration during training. A digital graphic scale ranging from 1 to 100 was provided for answering. The responses were evaluated via Pearson correlation analysis. Group differences were calculated using the Mann–Whitney *U* test. Subgroup analysis was performed via interaction analysis as described by Brankovic et al. 2019 [28]. Results were visualized using a rainforest plot showing the coefficient and the Standard Error (SE) of each interaction analysis as well as the *p* value for the interaction.

## Results

Data from 61 students were available for analysis. A detailed overview of baseline characteristics is provided in Table 1.

**Table 1:** Demographic data

	Control Group	SA-Group
	(*n* = 27)	(*n* = 34)
	Mean (standard deviation: SD)	Mean (SD)
General Data		
Age (in years)	23.1 (2.1)	24.0 (2.6)
Sex		
Male (in percent)	12 (44.4%)*	15 (44.1%)*
Female (in percent)	15 (55.6%)*	19 (55.9%)*
Dominant hand		
Right (in percent)	22 (81.5%)*	30 (88.2%)*
LEFT (in percent)	5 (18.5%)*	4 (11.8%)*
Semester	7.4 (1.5)	7.9 (1.5)
Experience in the OR		
Number of seen procedures (open)	30.7 (58.03)	24.5 (33.2)
Number of seen open cholecystectomies	0.2 (0.36)	0.9 (3.5)
Number of assisted procedures (open)	24.1 (53.63)	13.5 (28.7)
Number of assisted open cholecystectomies	0.04 (0.2)	0.2 (0.9)
Number of seen LCs	0.7 (1.7)	0.7 (2.1)
Personal characteristics		
Video games	0.4 (0.5)	0.3 (0.5)
Music instrument	0.3 (0.5)	0.4 (0.5)
Sport	0.4 (0.5)	0.4 (0.5)
Fine motor skills improving hobby	0.1 (0.3)	0.03 (0.2)
Attention-Tests		
Attention (self-assessment)	73.0 (15.6)	67.3 (15.7)
Video learning (self-assessment)	59.1 (14.1)	67.7 (16.5)
TMT-A (percent rank value)	78.4 (14.3)	76.5 (20.0)
TMT-B (percent rank value)	69.2 (28.3)	77.6 (18.3)
TMT (difference between A and B)	23.8 (16.6)	20.3 (8.4)
ZVT (percent rank value)	75.7 (18.9)	79.2 (17.4)
FAIR-L	66.5 (25.4)	80.6 (16.1)
FAIR-Q	46.7 (25)	54.7 (30.4)
FAIR-K	65.6 (25.8)	76.9 (22.1)
Pre-Test		
GOALS	18.7 (5.6)	18.6 (5.3)
Mistakes	4.4 (2.0)	3.9 (2.3)
OSATS	60.1 (11.9)	60.3 (11.8)
Time (in min)	71.4 (16.6)	68.5 (12.9)
Heidelberg VR Score	65.1 (14.9)	62.4 (13.1)

*Absolute (in percent)

### Primary outcome

The SA intervention resulted in a significant improvement in performance measured with the OSATS score (SA: 67.0 ± 11.5 versus control: 59.1 ± 14.0, *p* value = 0.034) as the primary outcome (Table 2). The optimized regression model shown in Table 2 included group, sex, age, dominant hand, ‘Heidelberg VR Score,’ OSATS-Baseline, VAS of the Post-Test gallbladder, video games, the self-assessed ability of learning from videos, the results of the attention tests (TMT-A and FAIR-q), as well as the number of already assisted LC.

**Table 2:** Linear regression model with post-test OSATS score as the dependent variable

		$\beta$-Coefficient	SE	*t* value	*p* value	95% confidence interval	
						Lower	Upper
(Intercept)		40.8	31.6	1.294	0.206	− 24.0	105.6
Group		**9.5**	**4.2**	**2.234**	**0.034**	**0.8**	**18.1**
Sex		0.4	5.0	0.089	0.929	− 9.6	10.5
Age		− 0.1	1.0	− 0.076	0.940	− 2.2	2.1
Dominant hand		6.2	7.1	0.872	0.391	− 8.4	20.8
Assisted LC (OR experience)		− 4.80	2.0	− 2.397	0.023	− 9.0	− 0.7
Playing of video games		5.9	4.2	1.410	0.169	− 2.7	14.4
Video learning (self-assessment)		0.2	0.1	1.634	0.113	− 0.1	0.5
TMT-A (percentile)		0.2	0.2	1.421	0.166	− 0.1	0.6
FAIR-Test (quality value)		0.1	0.1	1.281	0.211	− 0.1	0.2
Heidelberg VR Score		0.004	0.1	0.030	0.976	− 0.3	0.3
OSATS baseline		− 0.3	0.2	− 1.227	0.230	− 0.7	0.2
Visual analog scale (Difficulty of the LC)		− 1.0	1.4	− 0.770	0.448	− 3.8	1.7

Bold values indicate group significantly impacts performance

### Secondary outcomes

**Fig. 3:**
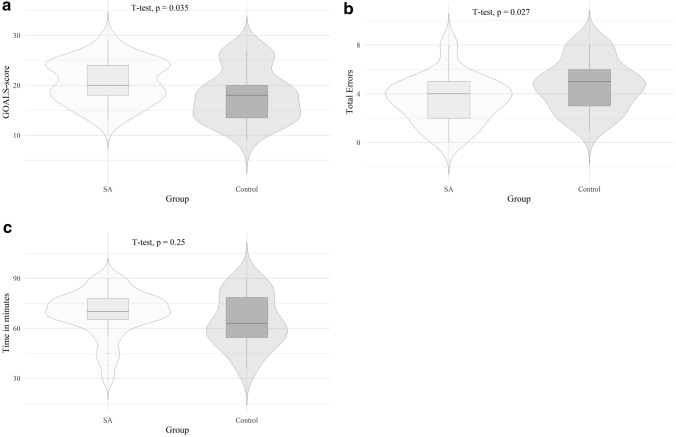
**a** Violin plot of group differences in GOALS-Score. **b** Violin plot of group differences in mistake-score. **c** Violin plot of group differences in time

The SA-group had significantly higher performance compared to the control group measured by the goals score (SA: 20.7 ± 4.3 versus Control: 18.0 ± 5.2, *p* value = 0.035, see Fig. 3a). The SA-group made significantly fewer errors than the control group measured with the error score (SA: 3.5 ± 1.9 versus Control: 4.7 ± 2.0, *p* value = 0.027, see Fig. 3b). The time in minutes to complete LC was the same in both groups. (SA: 68.7 ± 13.9 versus control: 64.2 ± 15.8, *p* value = 0.25, see Fig. 3c). Difficulties in the identification of important structures often led to their accidental severing. In the SA-group injuries of the artery or duct as well as clip misplacement occurred significantly less frequently than in the control group. There were no significant differences among both groups in preparation of the liver bed, liver injury, or in perforation of the gallbladder. These parameters were assessed with the error score (injuries of the artery or duct: SA: 44.1% no mistake, 38.2% minor mistakes, 17.6% major mistakes versus Control: 33.3% no mistake, 22.2% minor mistakes, 44.4% major mistakes; *p* value of the group difference = 0.069; clip misplacement: SA: 55.9% no mistake, 32.4% minor mistakes, 11.8% major mistakes versus Control: 25.9% no mistakes, 40.7% minor mistakes, 33.3% major mistakes; *p* value of the group difference = 0.034).

### Evaluation of the perceived usefulness of the SA intervention

**Fig. 4:**
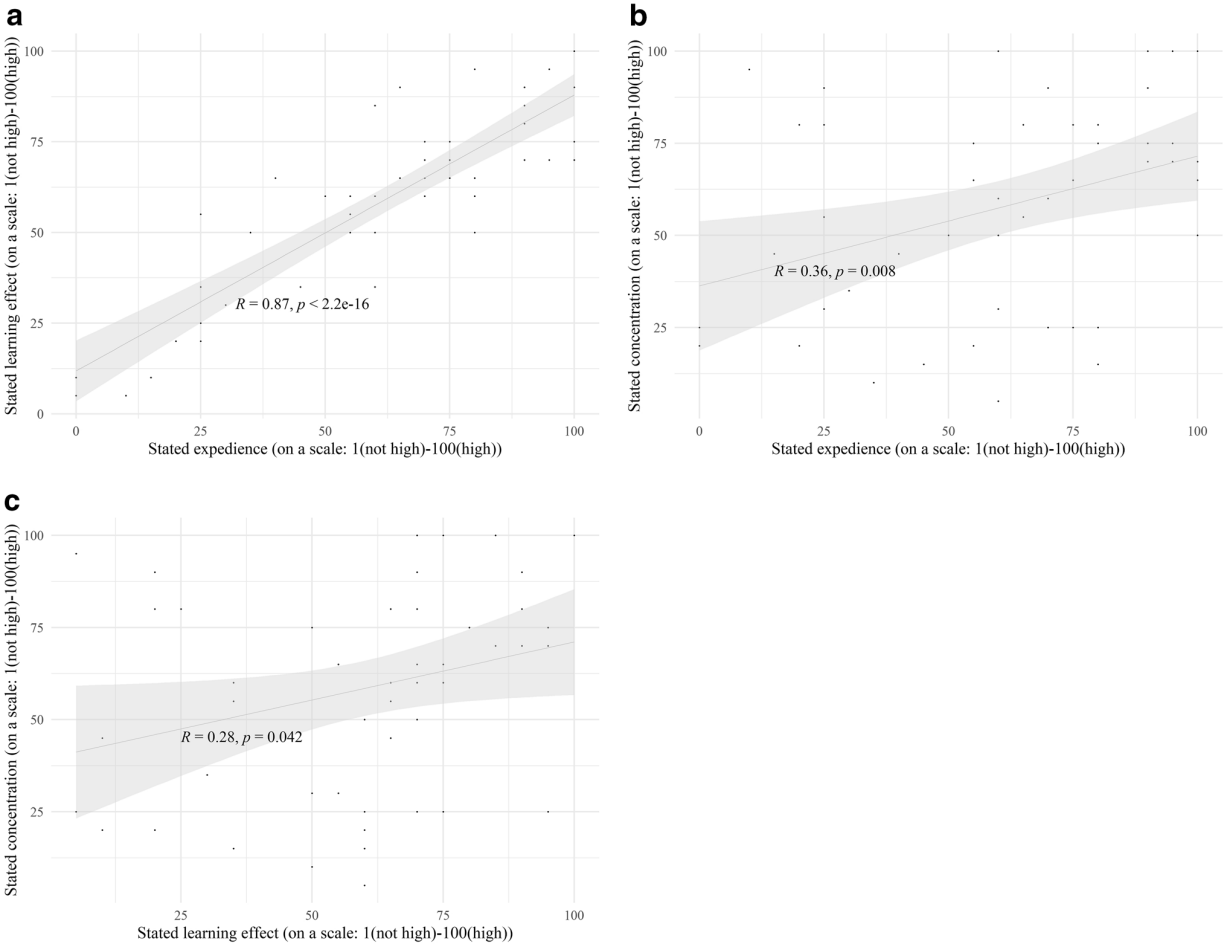
**a** Scatter plot showing the associations (Pearson correlations) between the stated learning effect and the perceived expedience of the cognitive training. **b** Scatter plot showing the associations (Pearson correlations) between the ability to concentrate and the perceived expedience of the cognitive training. **c** Scatter plot showing the associations (Pearson correlations) between the stated learning effectand the ability to concentrate of the cognitive training

Significant Pearson correlations appeared between the perceived learning effect and the perception of the perceived expedience of cognitive training (*R* coefficient = 0.87; *p* value < 0.001, see Fig. 4a). The stated expedience of the cognitive training and the stated ability to concentrate correlated significantly (*R* coefficient = 0.36; *p* value = 0.008, see Fig. 4b). The ability to concentrate and the stated learning effect also correlated significantly (*R* coefficient = 0.28; *p* value = 0.042, see Fig. 4c).

Looking at the evaluation questions of each individual group separately, no statistically significant differences were found (data not shown). However, 18 participants in the control group stated that they might benefit from the intervention; only two indicated that they might not.

### Subgroup analysis

Subgroup analysis did not reveal any unexpected adverse effects attributed to the intervention. Furthermore, no interaction between intervention and subgroup was detected (see supplementary file, Fig. 5).

**Fig. 5:**
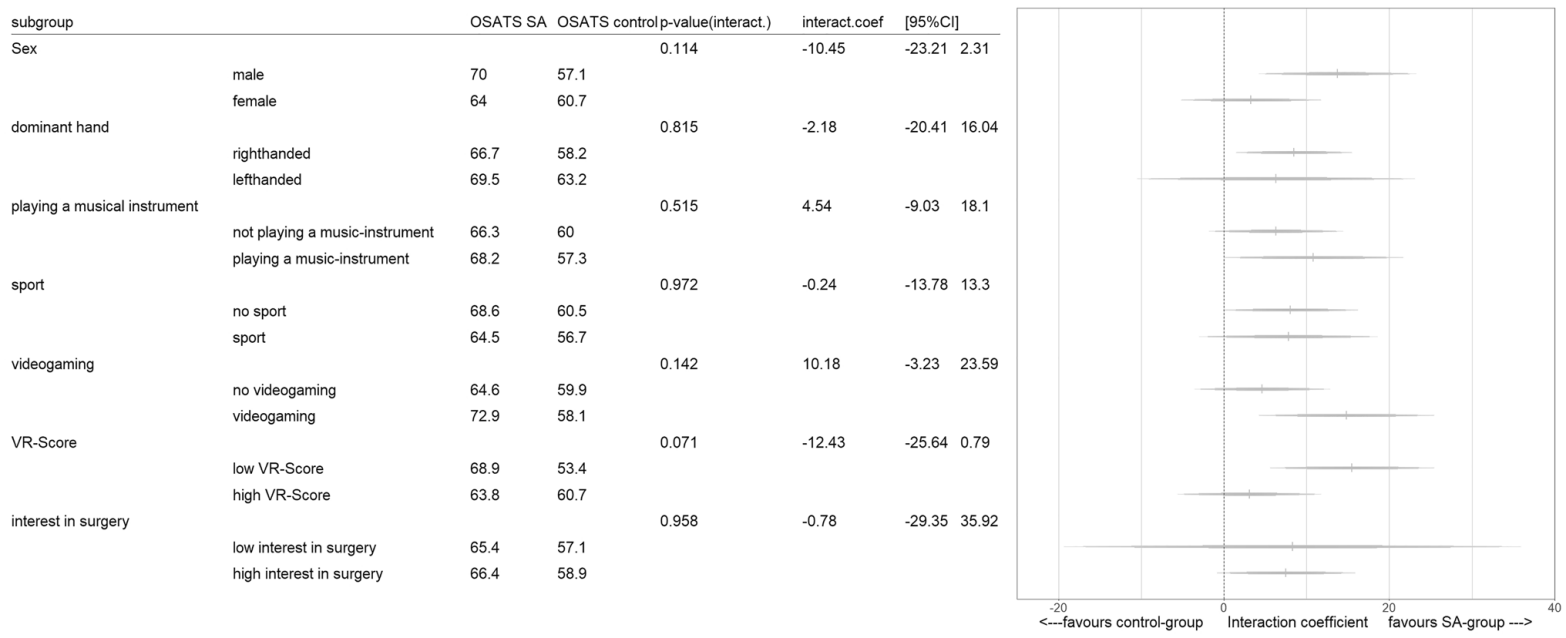
Rainforest plot illustrating subgroup analysis

## Discussion

The present randomized study shows that SA training as part of a multimodal training concept can lead to a significant improvement in laparoscopic performance during the early learning curve. The results were consistent in exploratory analyses for additional performance metrics and error rates. Significant group differences appear in favor of the SA-group for the OSATS score, the GOALS score, as well as the error score. These results imply that SA training helps to improve performance. Thus, the primary endpoint of this study was met. Due to the prospective randomized design and rigorous implementation, it can be assumed that the SA training is causal for the difference.

The finding that SA training as part of non-technical skills (NTS) training has a positive impact not only on cognitive but also on psychomotor skills is consistent with previous research [29, 30]. For example, McCulloch et al. stated that NTS training leads to improvements in team performance, error rates, and attitudes toward safety in LC. The ability of SA and its successful application is a contributor for surgeons to successfully perform a complex procedure. The surgeon must be able to recognize and react to intraoperative deviations such as anomalies of anatomy and physiology (e.g., altered coagulation parameters) and constantly question her/his own surgical performance to ensure patient safety. For example, by changing the surgical approach, communicating of abnormal coagulation to the anesthesiologist, or correcting a potentially problematic anastomosis. This is especially important for laparoscopic surgery. Most misinterpretations and thus errors occur under immense mental workload [16]. It becomes increasingly difficult to filter out relevant cues, while the actual focus is on the surgical procedure itself [31]. Cognitive training is well established in other fields, such as professional athletics and aviation. Also, medical studies revealed the benefits of cognitive training, especially in surgery. Mental training is associated with improved attention [32] and leads to better performance under stressful conditions. Anton et al. compared the outcome of suturing skills under induced stress between mentally trained and untrained residents and showed the superiority of the mentally trained group [33].

The intervention in the present study trained SA and thus an important cognitive function. It led to a reduction in errors in laparoscopic novices. The relationship between lack of SA and the occurrence of errors has been reported in the literature previously [34]. A systematic review by Graafland et al. showed the potential for error reduction in the OR through SA training. [16]. Looking more closely at the error score, the SA-group made significantly fewer errors in stump preparation, yet not in liver bed preparation. This indicates that the video clips targeting differentiation of artery, duct, and connective tissue had a positive impact. Difficulty in identifying the anatomical structures—artery, duct, or connective tissue—might lead to accidental dissection of the former two. Differentiation appears to have been easier for the SA-group than for the control group, as they made significantly fewer errors in dissecting the duct and artery. These results are analog to observations from other studies, suggesting that novices are likely to exhibit the greatest learning success in stump dissection [35]. The difference between stump and liver bed preparation has been investigated in another study using the GOALS score. The complexity of stump preparation in contrast to liver bed preparation was described as a possible reason [36].

However, despite displaying a superior performance, the SA-group took the same time as the control group to complete the LC. Graafland et al. showed a better response to equipment failure in the SA-trained group, but also found no difference in time required. McCulloch et al. observed an increase in NTS and attitude toward safety during LC, but no change in operating time after the surgeons participated for several months in aviation-style resource management training [17, 30]. Perhaps SA training led the SA-group to work more carefully and thus not improving in time despite better performance. This implies that SA training might shift the surgeon’s focus toward correctness and accuracy, i.e., the avoidance of errors and complications. Whether further experience in SA-trained surgeons leads to reduced procedure time while maintaining a low error rate remains to be elucidated in further studies. Also, the SA-group might have been less stressed than the control group. Pavlidis et al. hypothesized that a high stress level would be instrumental in working quickly in surgery; however, while hazarding an increase in likelihood of errors [37]. Both interpretations taken together conclude that SA training reduces the probability of errors without causing longer procedural time.

The evaluation questionnaire demonstrated that the video clips with SA training were rated more useful and led to higher concentration and learning effects than video clips without SA training. The correlations between the perceived subjective learning effect, level of concentration during training, and the expedience of cognitive training offer the conclusion that only those, who were able to concentrate on the video clips, stated a learning effect. These findings are in line with previous research stating that concentration and attention are crucial to learning effects [38].

The results of the subgroup analysis should be investigated in further studies as they only have explorative character. Various factors and traits influence the acquisition of laparoscopic skills, and this study indicates not all groups benefitted equally from SA training. Male and right-handed participants seem to have benefited from the SA training, but female and left-handed trainees did not benefit equally. Since the participants did not receive any instructions and female participants might perform worse than their male colleagues without instructions [39], it is reasonable to assume that the female participants were not able to realize their full potential. Not surprisingly, left-handers performed significantly worse in training designed for right-handers [40]. Therefore, both females and left-handers needed extra attention and extra working memory to compensate for their handicap. However, SA is highly dependent on attention and working memory [41].

In summary, SA training appears to be a useful complimentary tool to hands-on training in the multimodal training curriculum for novice laparoscopists. At this point, it has yet to be investigated whether SA training for LC can be applied to other, more difficult operations, and to what extent more difficult SA training sessions can provide more experienced surgeons with useful training. It is reasonable to assume that multimedia-based vicarious learning will be adopted by surgeons. A study shows the growing importance of collegial exchange via multimedia posts on social platforms, with a focus on learning through the mistakes and experiences of others [42]. Generally speaking, SA training is a promising opportunity to enhance the surgical skills and performance during early residency, possibly leading to more autonomy and motivation and thus positively affect the whole surgical team in addition to the patient’s safety.

Implementation of SA in the OR might be achieved through interceptive auto-suggestive questions the resident asks herself/himself while assisting and watching. Questions like “What’s going on at the moment?”, “What might happen next?”, and “Do I run into any risks soon?” help train SA. However, whether SA skills learned in a training center are applicable to the OR as well as the feasibility of its implementation and to what extent better SA means better outcomes in terms of mortality and morbidity for patients, needs to be verified in further studies. The feasibility of implementing this multimodal training concept can be difficult in smaller hospitals with limited resources. One way to overcome this problem is to have training centers at larger hospitals offer multimodal training for residents from smaller hospitals.

The integration of this study into the elective course came with limitations. Due to administrative reasons, the elective course was supervised by a total of two tutors and three assistants. Also, the MIS training center of the Surgical University Hospital Heidelberg is accessible and in use for various learning and exercise groups and it could not be guaranteed that only a single tutoring session took place at a given time. Despite previous arrangements, inter-rater validity and a generally valid schedule, individual differences in tutoring and distraction cannot be ruled out entirely. Another limitation of this study was that the participants were students. Medical students do not decidedly represent novice surgeons. However, all participants were novices and therefore comparable with each other. Equally limiting was the porcine cadaver liver model, from which no conclusions can be drawn as to whether the recorded increase in surgical performance is also associated with a benefit for alive human patients. On the other hand, the pig cadaver model is a well-established model for LC because of the similar anatomy, and it has already been shown that the technical skills learned can be transferred to humans in the operating room [43]. However, the ethical justifiability of porcine cadaver models is higher than that of living patients.

In conclusion, video-based SA training with a focus on potential errors during the critical steps of LC is beneficial in the early phase of laparoscopy training. Efficient training of novice laparoscopic surgeons is becoming increasingly important and SA training offers a valuable opportunity to add to multimodal training curricula. Video-based SA training could also be integrated into the concept of vicarious learning via social platforms and would thus be easily accessible. Further studies should focus on transferability to the OR and improved patient safety. Additionally, this is one aspect of a comprehensive training curriculum in addition to simulation and guided real cases.

## Supplementary Information

Below is the link to the electronic supplementary material.

Supplementary file1 (MP4 11248 KB)

Supplementary file2 (MP4 5251 KB)

## Abbreviations

GOALS: Global operative assessment of laparoscopic skills
IQR: Interquartile range
LC: Laparoscopic cholecystectomy
MIS: Minimally invasive surgery
NTS: Non-technical skills
OR: Operation room
OSATS: Objective structured assessment of technical skills
SA: Situation awareness
SE: Standard error
SD: Standard deviation
VAS: Visual analog scale
VR: Virtual reality
